# Use of an Orthodontic and Otolaryngological Approach in an Infant with Holoprosencephaly

**DOI:** 10.3390/children11050554

**Published:** 2024-05-05

**Authors:** Angela Galeotti, Giovanni Carlo De Vincentiis, Emanuela Sitzia, Giuseppe Marzo, Wanda Maldonato, Gaia Bompiani, Maria Beatrice Chiarini Testa, Alessandra Putrino, Andrea Bartuli, Paola Festa

**Affiliations:** 1 Dentistry Unit, Bambino Gesù Children’s Hospital, Istituto di Ricovero e Cura a Carattere Scientifico (IRCCS), 00165 Rome, Italy; 2 Private Practice, 00174 Rome, Italy; 3 Otolaryngology Unit, Bambino Gesù Children’s Hospital, Istituto di Ricovero e Cura a Carattere Scientifico (IRCCS), 00165 Rome, Italy; 4 Department of Life, Health, Environmental Sciences, University of L’Aquila, 67100 L’Aquila, Italy; 5 Pediatric Pulmonology & Respiratory Intermediate Care Unit, Sleep and Long-Term Ventilation Unit, Acdemic Department of Pediatrics (DPUO), Bambino Gesù Children’s Hospital, Istituto di Ricovero e Cura a Carattere Scientifico (IRCCS), 00165 Rome, Italy; 6 Rare Diseases and Medical Genetics Unit, Academic Department of Pediatrics, Bambino Gesù Children’s Hospital, Istituto di Ricovero e Cura a Carattere Scientifico (IRCCS), 00165 Rome, Italy; 7 Dentistry Unit, AORN Santobono-Pausilipon, 80100 Naples, Italy; paolafesta1@gmail.com

**Keywords:** holoprosencephaly, rare disease, infant, congenital nasal pyriform aperture stenosis (CNPAS), balloon dilation, orthodontics, palatal expander, craniofacial anomalies

## Abstract

Holoprosencephaly is a complex human brain malformation resulting from incomplete cleavage of the prosencephalon into both hemispheres. Congenital nasal pyriform aperture stenosis (CNPAS) is sometimes found in patients with mild forms of holoprosencephaly. Surgical treatment is required. Low-invasive surgical approaches involve balloon dilation of the pyriform opening. We present the case of an 8-day-old girl diagnosed with holoprosencephaly, CNPAS, and the presence of a solitary median maxillary central incisor. Once examined by neonatologist, geneticist, pneumologist, otolaryngologist, and pediatric dentist, a combined otolaryngological–orthodontic approach was used. The obstruction of the right nasal cavity was treated by widening the nasal cavities and stabilizing them with a balloon dilation technique. After surgery, the respiratory space was increased by applying a neonatal palatal expander plate (NPEP) considering the palatal deformity: ogival shaped, anterior vertex growth direction, reduction of transverse diameters. The NPEP promoted distraction of the median palatine suture and assisted the nasal dilation. Therefore, after the insertion of NPEP, the physiological sucking–swallowing mechanism was activated. In infants with CNPAS, NPEP can be useful to ensure the safe stability of nasal dilation. A multidisciplinary approach is fundamental. In our experience, the close collaboration between an otolaryngologist and orthodontist is essential for the management of the patient with CNPAS.

## 1. Introduction

Holoprosencephaly (HPE, MIM 236100) is a complex human brain malformation resulting from the incomplete cleavage of the prosencephalon into the right and left hemispheres, which occurs between the 18th and the 28th days of gestation [1]. The prevalence of HPE is around 1 in 10,000 births [2]. Approximately 25–50% of individuals with HPE have a chromosome abnormality, the most common being trisomy 13 [1,3]. Forebrain malformations are always associated with facial anomalies [1,4]. HPE is classified into different types based on the degree of severity. The most severe cases (alobar HPE) present cyclopia, anophthalmia, and synophthalmia, with or without a proboscis between the eyes, and are not compatible with life. Less severe cases (semilobar and lobar HPE) are often characterized by hypotelorism, a depressed nasal bridge, a prominent mid-palatal ridge, a midline cleft lip and palate, and the presence of a solitary median maxillary central incisor (SMMCI) [4,5]. Studies have reported an association between SMMCI and congenital nasal pyriform aperture stenosis (CNPAS) in patients with mild forms of holoprosencephaly [5,6]. In the case of HPE with SMMCI, the deficiency of lateral growth from the midline results in the premature fusion of the spreading dental laminae from the left and right sides in the maxilla. The inductive epithelium and mesenchymal condensations appear to merge, leaving the two distal halves of what would have been the two primary maxillary central incisors fused together, at this early stage of development, precisely in the midline. The permanent successor, arising from the palatal aspect of the primary tooth germ lamina, likewise forms a single symmetrical incisor of the permanent series and, again, consists of the distal halves of the permanent left and right central incisor teeth. The symmetry of the median-placed central incisors of both dentitions is virtually perfect [5,6]. CNPAS is a rare form of nasal airway obstruction caused by the overgrowth of the medial nasal process of the maxilla in the nasal opening. Since infants must breathe through the nose by up to 6–8 weeks of life, the increased resistance of the nasal airways due to the stenosis may cause a functional airway obstruction with consequent episodes of apnea and cyanosis [7,8]. Numerous treatments have been proposed for CNPAS, based on the severity of nasal respiratory failure. For mild cases, several studies recommend the use of nasal decongestants or corticosteroids, saline irrigations, anti-reflux drugs, humidifiers, oral breathing devices, or non-invasive positive pressure ventilation [9,10]. In the most severe cases, surgical correction of the stenosis is performed during infancy The traditional surgical technique is to perform an osteotomy of the lateral nasal wall followed by the placement of nasal stents, which are kept in place for 5–28 days [11]. Today, less invasive approaches involve balloon dilation of the pyriform opening, with or without nasal stent placement [12]. A recent paper reported the use of this new minimally invasive method (balloon dilatation) to achieve nasal patency, accompanied by the use of intraoral devices to promote and stabilize the dilatation [13]. The objective of this report is to present a clinical case of holoprosencephaly complicated by stenosis of the pyriform opening with acute respiratory failure and to propose a combined multidisciplinary otorhinolaryngology–orthodontic approach.

## 2. Case Report

**Figure 1:**
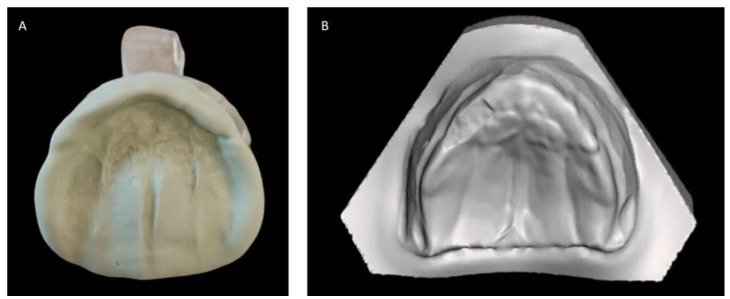
Dental impression of the maxillary arch in addition silicone material (**A**) and derived virtual plaster model (**B**).

**Figure 2:**
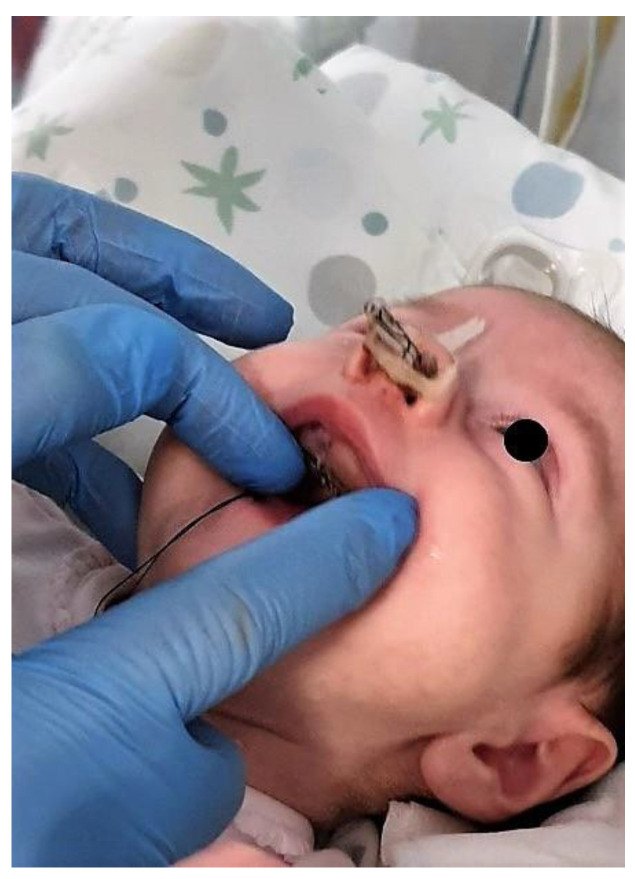
The patient at the time of the first application of the neonatal palatal expander plate (NPEP).

**Figure 3:**
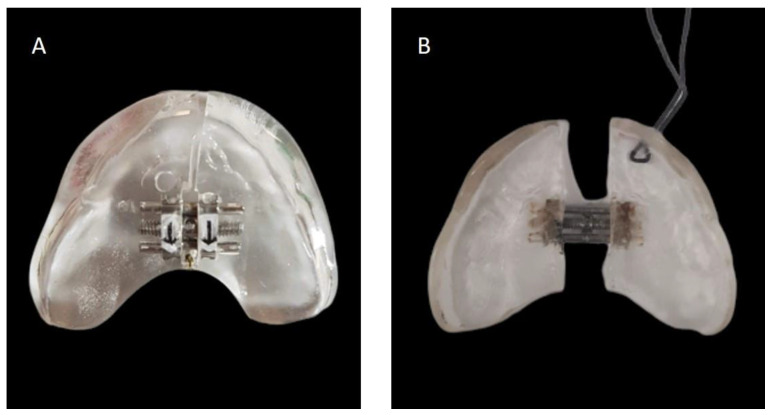
Appearance of the NPEP orthodontic appliance before activations of the central screw and insertion of the safety wire (**A**) and after the entire activation cycle (**B**).

**Figure 4:**
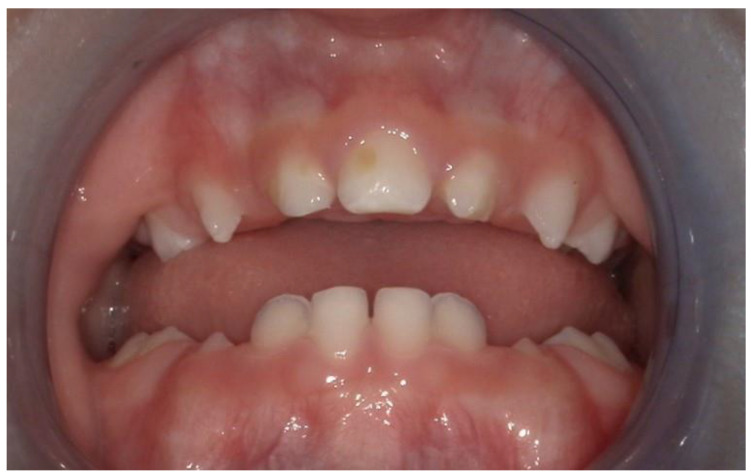
Two-year follow-up: the median maxillary central incisor is healthy and stable.

We present the rare case of an infant with HPE and CNPAS complicated by acute respiratory failure. The concomitant presence of an SMMCI was treated using a combined otorynolaringological–orthodontic approach. The multidisciplinary minimally invasive treatment involved a team comprising a neonatologist, a geneticist, a pneumologist, an ENT (ear, nose, and throat) doctor, and a pediatric dentist. The following is a detailed description of the events, the therapeutic path, and the long-term follow-up of the patient. An 8-day-old girl was admitted to the Neonatal Intensive Care Unit (TIN) of the Bambino Gesù Children’s Hospital (Rome, Italy). The baby was born at 39 weeks by caesarean section, with a birth weight of 2820 g and a length of 47.3 cm. The Apgar score was 8/10. During the clinical examination at birth, the following clinical signs were observed: cutaneous syndactyly of the II-III toes bilaterally, hypotelorism, low weight, and short stature. Respiratory distress and cyanosis ensued after a few hours. The patient was ventilated with neo-puff without much benefit. The infant was then administered CPAP (continuous positive airway pressure) and was intubated on the second day of life due to the worsening of her overall condition. Brain and facial mass MRI (magnetic resonance imaging) were performed at the birth hospital, revealing a congenital nasal pyriform aperture stenosis and the presence of a solitary median maxillary central incisor. On the eighth day of life, the patient was referred to the Bambino Gesù Children’s Research Hospital where she underwent the following tests: abdominal ultrasound, eye examination with negative results, GH, FSH, LH, cortisol, thyroid hormones, and ACTH levels, the results of which were all in the normal range. The infant also underwent an echocardiogram, which showed evidence of pervious foramen ovale. After evaluation at the Medical Genetics Department, molecular analysis showed a heterozygous variant of the GLI2 gene, for holoprosencephaly, which is classified as a class IV variant arising de novo, and which was absent in the DNA extracted from the blood of the parents. Pathogenetic variants of the GLI2 gene are associated with an autosomal dominant form of holoprosencephaly (HOLOPROSENCEPHALY 9; OMIM # 610829) with variable phenotypic expression and, in some cases, incomplete penetrance [14]. The infant was then evaluated by the ENT doctor, who performed a nasal endoscopy using a flexible laryngoscope under general anesthesia, which revealed the presence of mesenchymal bridges obstructing the right nasal cavity and a left-convex dislocation of the nasal septum. After this evaluation, the ENT doctor decided to proceed with the lysis of mesenchymal bridges, and subsequently a gentle dilation of the nasal cavities (Hegar of increasing caliber up to 4) was performed. After the widening of the nasal cavities, stabilization was achieved through balloon dilation, and two trans-nasal stents with small calibers (2.5 in one nasal cavity and 3 in the other) were placed. The infant was simultaneously evaluated by a pediatric dentist who specialized in orthodontics. The dentist found evidence of the following clinical signs: the palate had a markedly ogival shape, with growth in the direction of the anterior vertex; the median raphe palatine was severely depressed; and there was a reduction in the transverse diameters of the upper jaw. In the operating room, the dental team took an impression of the palate with addition silicone in order to create an intraoral device (Figure 1). After the surgery, given the presence of the characteristic palatal deformity, a decision was made to increase the respiratory space by applying a device. On the same day, a neonatal palatal expander plate (NPEP) [13] was built by a dental technician and positioned in the upper arch (Figure 2). The device was a mucous anchoring plate made of acrylic resin, which was extended buccally with flanges to ensure good retention [13]. A screw was placed at the center of device, to correspond with the palate median suture. To avoid any risk of suffocation, the team inserted a safety wire through two holes in the canine region. The safety wire was a surgical silk suture thread (without a needle) of approximately 70 cm in length. The orthopedic maxillary expansion therapy was initiated in order to favor the distraction of the palate at the level of the median palatine suture and assist the nasal dilation. A precise protocol for the use of the orthodontic device was established, and the child responded very well. The saturation and frequency remained regular during the insertion maneuvers, and the child kept the device in her mouth throughout the procedure. We recommend activating the central screw of the device 24 h after insertion (Figure 3). In this case, for the first day, the infant wore the device for two hours, three times a day. On the subsequent days, the device was worn for three hours, three times a day, under the constant supervision of the parents or caregivers. Immediately after the insertion of the device, the patient started swallowing and sucking normally, demonstrating that the physiological sucking–swallowing mechanism had been activated. Five days after surgery, the stents were removed and the girl was able to breath on her own, with a few episodes of desaturation that spontaneously resolved. Subsequently, the clinical condition of the child remained stable, and the follow-up visits with the ENT specialists and the orthodontist showed positive results: she resumed spontaneous breathing with good saturation (97%), her bottle feeding was going well and she had gained weight, and a valid nasal respiratory space had been secured, which was associated with the rosy appearance of the soft tissues of the oral cavity. After 15 days, the use of the expansion device was suspended and the patient was discharged. The patient underwent follow-up visits once a month for the first six months and thereafter every six months for the monitoring of respiratory function and airway patency, dental eruption, the stability and health of the median maxillary central incisor, and the growth of the jaw complex (Figure 4).

## 3. Discussion

Holoprosencephaly (HPE) is one of the more common malformations of the brain and face in humans and represents a wide clinical spectrum of disorders ranging from simple features, such as a single central maxillary incisor or closely spaced eyes, to extreme forms like a single cyclopic eye and superior proboscis [14]. HPE is deemed by most researchers to be caused by the genetic loss or mutational dysfunctions of minimal critical regions and relatively rare cytogenetic rearrangements that serve as core genetic susceptibility factors for humans in different risk factor genes at each key locus: SHH at 7q36, SIX3 at 2p21, ZIC2 at 13q3.2, and TGIF at 18p11.3 [15]. When examined carefully, mutations in these or related genes have been shown to result in proteins with diminished biological function: SHH, SIX3, GLI2, TGIF, ZIC2, DISP, and the NODAL pathway [16]. Recently, gene–environment interactions have been evaluated and the risk and/or severity of HPE has been linked with maternal pregestational diabetes, alcohol, folic acid, allergies, consumer products, including foods rich with cholesterol in vitro fertilization, and X-rays [17]. Accurate prenatal evaluation is important because an in vivo diagnosis can be established using magnetic resonance imaging (MRI), and the severity of imaging findings is correlated with postnatal morbidity and mortality in HPE. This early diagnostic approach is highly useful for the evaluation, management, and genetic counseling that families can receive during a pregnancy [18]. In the clinical case described, the diagnosis, based on the patient’s clinical condition, was postnatal thanks to MRI and genetic analysis. This avenue of diagnosis is crucial given the heterogeneity of the disease, which, in a more severe form, would have been intercepted during pregnancy. In accordance with the literature [19] and based on our experience, the provision of information and support to families experiencing HPE in a loved one is unequivocally challenging. Pertinent information and medical guidance, which serve as the foundation for shared decision-making, are key to providing “personalized medicine” to the families of patients affected by HPE that we encounter. This is also fundamental with the less common varieties, like the one we reported, since their recognition in the second trimester can be quite difficult compared to the severe varieties that are easily detected during first-trimester screening [20]. As expected, a great deal of parental anxiety is associated with HPE as there is no clear prognosis concerning the condition. While severe cases of HPE are associated with high rates of early mortality, children with milder forms can survive beyond infancy [20,21]. However, survival is associated with facial anomalies, hydrocephalus, seizures, motor impairment, hypothalamic or endocrine dysfunction, and gastrointestinal and respiratory problems. Genetic studies, karyotyping, FISH, and/or syndromic specific testing (including isolated HPE genetic testing) are useful in determining the key role of a specific familial genetic or chromosomic abnormality and in predicting the risk in future pregnancies. Children surviving with HPE require a multidisciplinary approach and management from different medical and surgical specialties, as well as rehabilitation to ensure successful patient care and parental support [19,21]. One of the most frequent anomalies associated with HPE is the median maxillary single central incisor (SMMCI) and congenital nasal pyriform aperture stenosis (CNPAS) [22,23]. The diagnosis and management of clinical cases with this association are described in the literature [6,7,24,25,26,27,28,29,30,31,32,33] (Table 1). From the earliest to most recent studies, such as our case, the diagnosis of minor forms of HPE has been reported to occur after birth, mainly due to respiratory difficulty and the need for assistance (intubation in intensive care). Imaging tests such as CT and MRI help to evaluate the presence of nasal obstruction [6,7,22,23,24,25,26,27,28,29,30,31,32,33]. The presence of SMMCI is often incidentally detected [6,7,24,25,26,27,28,29,30,31,32,33]. Over the years, the diagnosis has been increasingly supported by postnatal genetic analyses, and the current trend strongly encourages the evaluation of risk factors during pregnancy and early diagnosis as early as the fetal period so that the necessary postnatal approach can be planned [18].

The treatment of stenosis of the pyriform opening associated with holoprosencephaly can be limited to medical therapy for mild cases [6,25,26,29,33]. However, the most severe cases require correction of the anatomical defect with surgery [7,24,27,28,29,30,31,32]. In the traditional approach, surgery is followed by the placement of nasal stents to stabilize the dilatation [7,24,27,28,29,30,31,32]; despite the effectiveness of this approach, it can lead to several post-operative complications, like restenosis, nasolacrimal duct injury, anomalies of the facial mass development, and abnormal dentition [34]. The balloon dilation approach, on the other hand, represents an innovative and minimally invasive method that can be performed with or without the placement of nasal stents [12]. The plasticity of the bone in infants enables the immediate resolution of the stenosis, even if a risk of restenosis has been indicated [35]. Thus, nasal stents can be used to stabilize the dilation, although frequent damage to the nasal mucosa has been reported [15]. Therefore, new alternatives might be considered to stabilize the results. The approach involving a rapid palate expander is frequently used in children from four years of age to increase the transverse diameters of the palate, widen the floor of the nasal cavities, improve the patency of the upper airways and enhance respiratory function in cases of obstruction [35,36]. A different orthodontic approach, for children of the same age, entails the use of a quad-helix to obtain an early expansion followed by a second step of expansion with the same appliance after eruption of the permanent dentition is complete, combined with the backwards movement of the posterior teeth and opening space with fixed multibracket therapy for the missing central incisor (prosthetically added) [31]. Ruling out or confirming the presence of syndromic or non-syndromic dental abnormalities to determine the best direction for early orthodontic approaches will improve the developmental prognosis of the young patient if correctly diagnosed and fully understood [37,38]. Recent experiences support the advantage of using orthodontic devices with newborn patients affected by syndromic or non-syndromic abnormalities involving oral structures [39,40]. From a diagnostic standpoint, the condition treated in this case could hardly have been misdiagnosed, but it was the contribution of all of the involved specialist skills that made the difference. Therefore, we suggest that the use of NPEP in infants with CNPAS can help ensure the stability of the dilation, without complications. Indeed, NPEP represents a safe, non-invasive, and totally reversible approach. Taking dental impressions while the patient is under general anesthesia is ideal since the materials do not introduce any risk of suffocation and the device has excellent grip and stability as it exploits the undercuts present especially at the pre-maxilla level. Moreover, the device can be immediately removed by the medical staff or the caregiver should any adjustments or changes ever been needed, based on the possible rapid growth of the child or other problems. It is essential to educate the parents, the caregiver, and medical staff on how to insert, activate, and remove the device. Insertion should occur within a minimum of 24 h and a maximum of 48 h after the realization of the impression to avoid problems with device retention due to the rapid growth of the infant. Additionally, the stability of vital parameters should be monitored continuously. After 15 days, the obtained expansion should be stable and the treatment must be stopped, to avoid interference with the physiological growth of the craniofacial region. Also of interest was the patient’s reaction to the insertion of the device. She immediately activated the physiological mechanism of sucking, swallowing, and breathing. From the 13th week of intrauterine life, a fetus can swallow and, at the 5th month, it can suck its thumb [41]. Sucking is a complex neuromuscular activity that is regulated by a bulbar center, whose activity is synchronous with that of the breathing center. At birth, there is a 1:1 ratio between sucking and swallowing, while breathing occurs between one swallowing act and the next [42]. In newborns, there are three important acts to coordinate: sucking, swallowing, and nasal breathing [43]. An imbalance caused by mechanical obstruction of nasal breathing negatively affects the entire mechanism of sucking–deglutition–breathing [31]. Therefore, we hypothesize that in the case described above, the intervention of nasal dilation resulted in the patency of the airway, and that the insertion of NPEP into the oral cavity induced a new higher position of the tongue and swallowing mechanism. Moreover, the correct positioning of the tongue and the activation of the entire mechanism of sucking–deglutition–breathing could have allowed the development of the upper jaw in a transverse direction. Thus, it is crucial to monitor the patient’s craniofacial growth over time as a new maxillary expansion treatment may later be needed. Furthermore, the presence of the SMMCI, as reported in previous research, could significantly influence the arch form, in the direction of the anterior vertex, and the development of the maxilla–mandibular complex [32]. Concerning the possible limitations of our study, we hope to document the evolution of the patient’s clinical conditions with a more detailed follow-up over time. With a few exceptions [31], the criticality of the cases documented in the literature is reflected in the follow-up. However, often, emergency management and clinical checks are only documented up to around the third year of life. Subsequent evaluations help to provide a broader vision of the anatomical–functional evolution of these conditions and their improved management from the moment of diagnosis and onward as the patient grows.

## 4. Conclusions

In this case, the multidisciplinary approach involving a pneumologist, geneticist, ENT doctor, neonatologist, and pediatric dentist was fundamental. In particular, the close collaboration between the ENT doctor and orthodontist was essential for the management of this patient with congenital nasal pyriform aperture stenosis. The nasal patency ensured by the surgical procedure and the maxillary expansion provided interesting results in terms of good stability and dimensions and palate width. We conclude that this approach may be considered as a treatment for infants with holoprosencephaly.

## Figures and Tables

**Table 1:** Previous clinical cases published in the literature reporting an association between at least two of these conditions, HPE, CNPAS, and SMMCI, and describing the diagnosis and management.

Authors and Year	Sample Size, Anomalies, and Features Observed	Diagnosis	Management
Royal et al., 1999 [24]	2 cases of CNPAS and SMMCI	CT at birth (30 week of gestation) and at 4 months of life for significant nasal obstruction and respiratory distress	Both patients were intubated and, subsequently, when they became stable, they started a medical therapy with a nasal beclomethasone spray. Both patients failed this conservative approach and they underwent surgical procedures and stent applications.
Chan et al., 2005 [6]	1 case of HPE with CNPAS and SMMCI	CT at birth (17 days old) for respiratory distress, followed by chromosome study	After intubation for 8 days, the parents opted for non-surgical management due to cerebral malformation and poor neurodevelopmental prognosis. To relieve the nasal obstruction, 1.5% sodium chloride nasal drops were given four times per day and we started stenting her nostrils alternatively with an endotracheal tube daily. The size of the stenting tube was gradually increased from 2 mm (outer diameter 2.9 mm) to 3.5 mm (outer diameter 4.8 mm) over a period of 55 days. The oropharyngeal airway was taken off on day 65 of life successfully. She had satisfactory weight gain afterwards. At 15 months old, her body weight was in the 50–75th centile and her height was in the 25th centile. Although she was well on room air, she may have been at risk of sleep apnea.
Levison et al., 2005 [25]	2 cases of CNPAS and SMMCI (of which 1 case was with HPE)	CT and MRI at birth and 2 weeks of life, respectively, due to respiratory distress and nasal obstruction	The patients were treated with size 5 Fr gauge catheters passed nasally but with difficulty. An axial CT scan of the midface showed CNPAS and an SMCI. A cranial magnetic resonance imaging (MRI) scan showed the SMCI with a normal brain anatomy, including demonstration of the pituitary gland. A karyotype, baseline pituitary hormone and glucose tests, and ophthalmic assessment were normal. In both the cases, the nasal obstruction improved transiently with topical steroid drops, but one of the babies needed surgical enlargement of the bony nasal aperture, via a sublabial approach, at 4 months of age due to continued feeding difficulties.
Tagliarini et al., 2005 [26]	1 case of HPE with CNPAS and SMMCI	CT at birth for respiratory distress	The patient underwent surgical correction and a sylastic nasal splint to prevent the formation of adherences. On the fifth post-operative day, the nasal splint was removed and we used weekly dressings with good evolution. The child presented improved nasal breathing, with consequent weight gain, and she presented normal development at the age of 3 years, with physical and facial growth within the normal range. Deciduous teeth grew without abnormalities. Control paranasal sinuses CT scans were conducted at 7 months and 2 years to demonstrate good development of nasal fossae, even though she still had partial narrowing of the middle third, in addition to the presence of dental germens of central incisors and an absence of associated malformation. The child was followed up until the age of 3 years.
Devambez et al., 2009 [27]	21 cases of CNPAS and SMMCI	CT at birth (age ranging from 0 to 88 days) for poor nasal respiration or severe neonatal respiratory distress (9 cases)	Initial treatment was based on humidification, topical nasal decongestants (including epinephrine drops), treatment of gastroesophageal reflux, with or without an oral cannula (Guedel or Mayo), and enteral feeding via a nasogastric tube. If there was no significant clinical improvement after 7 to 10 days, a surgical procedure was proposed, except in the case of subtotal nasal obstruction observed on a CT scan, which required an earlier surgical intervention.
Blackmore et al., 2010 [28]	1 case of CNPAS and SMMCI	CT at birth for respiratory distress	Failed nasal intubation led to nasoendoscopy, which suggested a diagnosis of unilateral choanal atresia. Orogastric feeding was commenced but converted to total parenteral nutrition due to poor absorption and high stomal output. She was extubated for several days but developed worsening respiratory distress secondary to thick nasal secretions. As a result of her increased respiratory effort and carbon dioxide retention, the decision was made to proceed to surgery to correct the nasal abnormalities on day 28. Stents were left in place for 4 weeks. Post-operatively, her respiratory state returned to normal.
Thomas et al., 2010 [29]	1 case of CNPAS with SMMCI	CT confirmed the clinical suspicion of CNPAS in a 30-day-old baby	There was a history of respiratory distress and cyanosis at birth. A flexible nasopharyngolaryngoscopy was attempted, but the scope could not be negotiated toward the choanae. A No.6 nasogastric tube also could not be passed through the nostrils. After the CT, the child improved symptomatically with conservative measures such as insertion of an oral airway and feeding in the upright position. No active intervention was undertaken during this visit and the patient was asked to report for review early, in case there was any symptomatic worsening.
Visvanathan et al., 2012 [30]	10 cases of CNPAS and SMMCI	CT confirmed the clinical suspicion of CNPAS in all the cases (and SMMCI in 5 cases)	The position of the NP tube was checked with a nasendoscope and tube care included regular nasal suction and decongestants. Persistent airway obstruction despite NP tube insertion was an indication for surgery. The definitive treatment was surgical in five children and medical in five cases. Medical management included nasal decongestants, humidification, nasopharyngeal airway insertion, and management of laryngopharyngeal reflux.
Lygidakis et al., 2013 [31]	1 case of SMMCI and CNPAS (14-year follow-up and orthodontic treatment)	Radiographic findings at 4 years old for dental treatment	The medical history indicated respiratory distress and surgery soon after birth due to CNPAS. Gradual orthodontic treatment was started at the age of 4 years and completed at the age of 13 years. Following maxillary expansion, upper lateral segments were moved backwards and anterior space was created to accommodate a second central incisor. Retainers with a supplementary acrylic incisor were provided for aesthetic and functional replacement until the age of 16 years, when a fixed Maryland ceramic bridge was placed. Two-year recall, at the age of 18 years, revealed a satisfactory and stable aesthetic and functional result.
Moreddu et al., 2016 [32]	10 cases of CNPAS, 5 of which were with SMMCI	All children underwent a craniofacial CT scan to confirm the diagnosis	Medical treatment with nasal saline and decongestants (four drops in the nostril of 10% adrenaline saline) was first performed. Persistent airway obstruction symptoms despite this treatment were an indication for surgical intervention. All operated patients underwent the same surgical procedure. Stenting of the nasal fossae was performed using Portex 3.0 “blue line” endotracheal tubes for a maximum of four weeks. Post-operative stent care consisted of a normal saline nasal wash and nasal decongestion using four drops of the 10% adrenalin saline mixture per nostril three times a day.
Serrano et al., 2016 [7]	2 cases of CNPAS, 1 of which was with HPE (and SMMCI)	CT at birth for respiratory distress and surgical correction (the patient, affected by HPE, remained dependent on tracheostomy)	The patient in the first case had no concomitant comorbidities, and the outcome was successful after surgical correction of stenosis. The patient in the second case had an associated holoprosencephaly, and although surgical correction and nasal cavity patency were achieved, the patient remained dependent on tracheostomy due to dysphagia and neurological impairment.
Ilhan et al., 2018 [33]	1 case of HPE with CNPAS and SMMCI	CT and genetic analysis at postnatal 64 days in a patient born at 32 weeks of gestation	The patient at birth underwent intubation and mechanic ventilation, and after 64 days, a 5-French nasogastric tube was forced, advanced through both nostrils. The patient had received hydrocortisone, levothyroxine, and desmopressin in the referring center. After the CT and genetic analysis, an otorhinolaryngologist and a neurosurgeon were consulted, and no surgical intervention was considered. Nasal decongestants and dexamethasone-containing nasal drops were used for conservative purposes for 15 days. The patient was evaluated for future anomalies that could develop during dentition and referred to an orthodontics clinic. As the patient was unable to feed orally, the parents were trained on feeding with an orogastric tube and the patient was discharged at postnatal day 81.

## Data Availability

The data presented in this study are available on request from the corresponding author. The data are not publicly available due to privacy.

## Abbreviations

HPE: Holoprosencephaly; SMMCI: Solitary Median Maxillary Central Incisor; CNPAS: Congenital Nasal Pyriform Aperture Stenosis; TIN: Neonatal Intensive Care Unit; CPAP: Continuous Positive Airway Pressure; MRI: Magnetic Resonance Imaging; NPEP: Neonatal Palatal Expander Plate; ENT: Ear, Nose, and Throat.
